# Effect of pharmacist-led inhaler technique assessment service on readmissions in hospitalized COPD patients: a randomized, controlled pilot study

**DOI:** 10.1186/s12890-022-02004-z

**Published:** 2022-05-27

**Authors:** Adyam Tesfamariam Kebede, Elin Trapnes, Marianne Lea, Bjørg Abrahamsen, Liv Mathiesen

**Affiliations:** 1 Department of Pharmacy, Section for Pharmacology and Pharmaceutical Biosciences, University of Oslo, Oslo, Norway; 2 Department of Pharmaceutical Services, Oslo Hospital Pharmacy, Hospital Pharmacies Enterprise, South-Eastern Norway, Oslo, Norway; 3 Chief Physician at the Department of Pulmonary Medicine, Oslo University Hospital, Oslo, Norway

**Keywords:** COPD, Inhaler technique, Pharmacist-led training, Hospital readmissions CAT-score

## Abstract

**Objective:**

To investigate the effect of pharmacist-led inhaler technique assessment service on readmissions and CAT-score in hospitalized COPD patients. Furthermore, to provide an effect estimate for sample size calculations for future studies and to gain experience on the feasibility of such studies.

**Methods:**

A randomized controlled pilot study. Patients were randomized 1:1 to intervention or standard care. The primary endpoint was the difference in time to first readmission after hospital discharge between the treatment groups.

**Results:**

There was no statistically significant effect on the time to readmission (median 41 days in the intervention group (19 patients) and 95 days in the control group (20 patients), HR 1.74, 95% CI 0.81–3.75, *p* = 0.16). There was no statistically significant difference between the groups in CAT-score 2 months after discharge, median scores being 25.5 and 24 in the intervention and the control group, respectively (*p* = 0.29). There was, however, a reduction of 3.5 units in CAT-score from baseline to 2 months after discharge in the intervention group, compared to no change in the control group.

**Conclusion:**

Pharmacist-led inhaler technique training had no effect on time to readmission or CAT-score. Future studies in larger populations should consider focusing on patients with less severe COPD, exploring CAT-score as a primary endpoint, consider stratifying for important baseline variables and evaluate the acceptability of the intervention.

***Trial registration*:**

Date of registration 01/10/2018. ClinicalTrials.gov identifier: NCT03691324.

## Introduction

Chronic obstructive pulmonary disease (COPD) is characterized by an obstruction of lung airflow that interferes with normal breathing [1]. COPD is a leading cause of morbidity and mortality worldwide [1–3], and is the most frequent cause of hospital readmissions along with heart diseases [4, 5]. COPD exacerbations are acute worsening of respiratory symptoms often requiring hospitalization and accounting for the greatest proportion of the total burden of COPD [1, 6, 7]. The COPD Assessment Test (CAT) is a scoring system that assesses the impact of COPD on the patient's health, and can be employed as a predictor of severity of airway obstruction [8, 9].

Inhaled drugs are central to the therapy of COPD, aiming at improving symptom control and reducing the frequency and severity of exacerbations [1, 10, 11]. Inhaled drugs have the advantage of the active substance depositing in the target organ, allowing the use of lower doses compared to systemic delivery [12]. The lower systemic drug concentration leads to fewer and less severe adverse effects [12, 13]. Another advantage is that the drug is not exposed to barriers to therapeutic efficacy, such as poor gastrointestinal absorption and first-pass metabolism [14, 15].

Errors in the use of inhalers are common among both new and experienced patients [16–19]. There is a range of inhaler devices and patients may use several devices that require different techniques [20–22]. Furthermore, each type of device has several technical steps that must be conducted correctly for optimal effect [23]. Patients might risk a sub-optimal effect of their inhaled drug due to incorrect use [19–21, 24–26], leading to inadequate symptom control, poor health outcomes, and an increased risk of exacerbation and hospitalization [17, 27–32]. Previous studies have shown that patient education can improve the inhaler technique [33–41]. Although inhaler technique training has been shown to be associated with reduced exacerbation rates in hospitalised patients with obstructive lung diseases, the studies had some limitations [34, 40]. Song et al. included only patients using metered dose inhalers [34]. The meta-analysis by Maricoto et al. included eight studies about the effect on clinical outcomes, including exacerbation rates, but the interventions were carried out in different settings and there was a high discrepancy in the reported results [40]. Thus, there is only a limited number of studies, with varying quality, exploring the effect of inhaler technique training on clinically relevant endpoints like the CAT-score or hospital readmissions [42–44].

The main objective of the current study was to provide an effect estimate of an educational inhaler technique intervention on time to first readmission, and on CAT-score 2 months post-discharge. The effect estimate would provide information into sample size calculations for the planning of future, confirmative randomized controlled trials. Furthermore, we aimed to gain experience with respect to the feasibility of such studies, including aspects such as recruitment rate and the patients’ acceptability of the intervention.

## Methods

### Study design

This is a randomized, controlled pilot study, to which patients were allocated (1:1) to the intervention or the control group. Patients were considered for inclusion Monday to Friday during daytime shifts and were recruited between September 26th 2018 and January 31st 2019 from The Department of Pulmonary Medicine at a hospital in Oslo, Norway. Ethics approval was obtained from the Norwegian Regional Committees for Medical and Health Research Ethics (2014/704/REK South-Eastern Norway) and the hospital’s Privacy Ombudsman. ClinicalTrials.gov identifier: NCT03691324, registration date 01/10/2018.

### Participants and enrolment

**Fig. 1:**
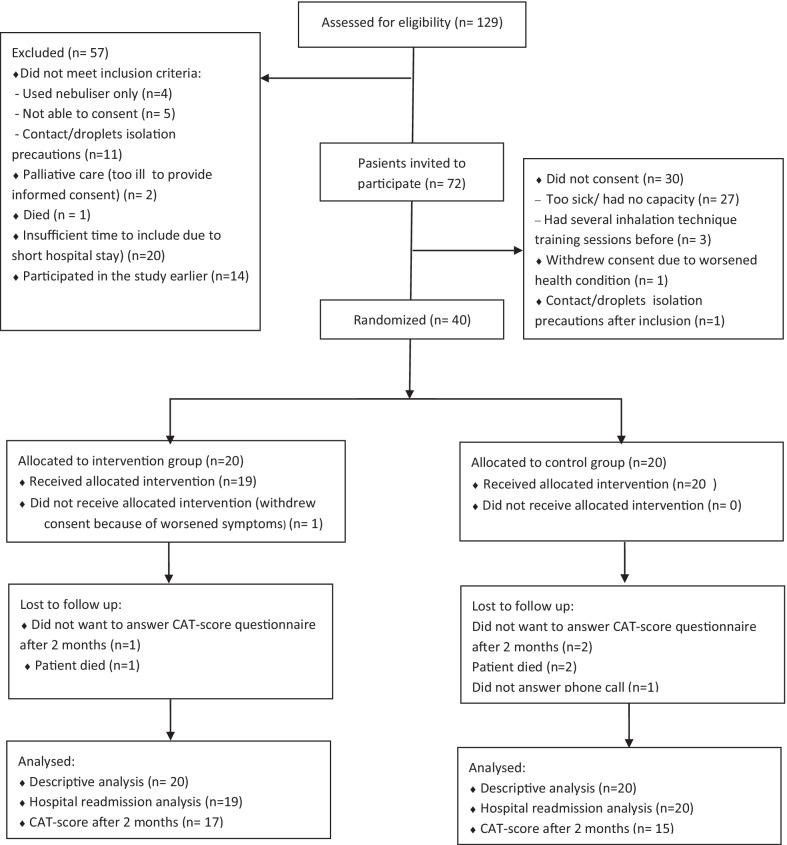
CONSORT flow chart showing the flow of patients throughout the pilot study

Patients hospitalized at the ward were eligible for inclusion based on the following criteria: diagnosed with COPD, using an inhaler for COPD treatment, administering their own inhalers and willing and able to provide written informed consent. Patients using nebulizers or being at risk of transmitting infections, thus imposing strict isolation procedures, were not eligible for inclusion. Patients readmitted during the study period were not invited for a second inclusion. Two master students in pharmacy recruited patients and collected data. Eligible patients were approached preferably within 24 h after hospital admission (Fig. 1).

### Baseline assessments

Baseline data were collected from patient interviews using a data registry form based on the official national Norwegian COPD-registry form, the hospital record, and the national summary care record [45], and included age, sex, comorbidities, COPD stage, inhaler medication(s) in use, the number of admissions by any cause, and COPD-related admissions to the hospital during the last 12 months prior to inclusion.

**Fig. 2:**
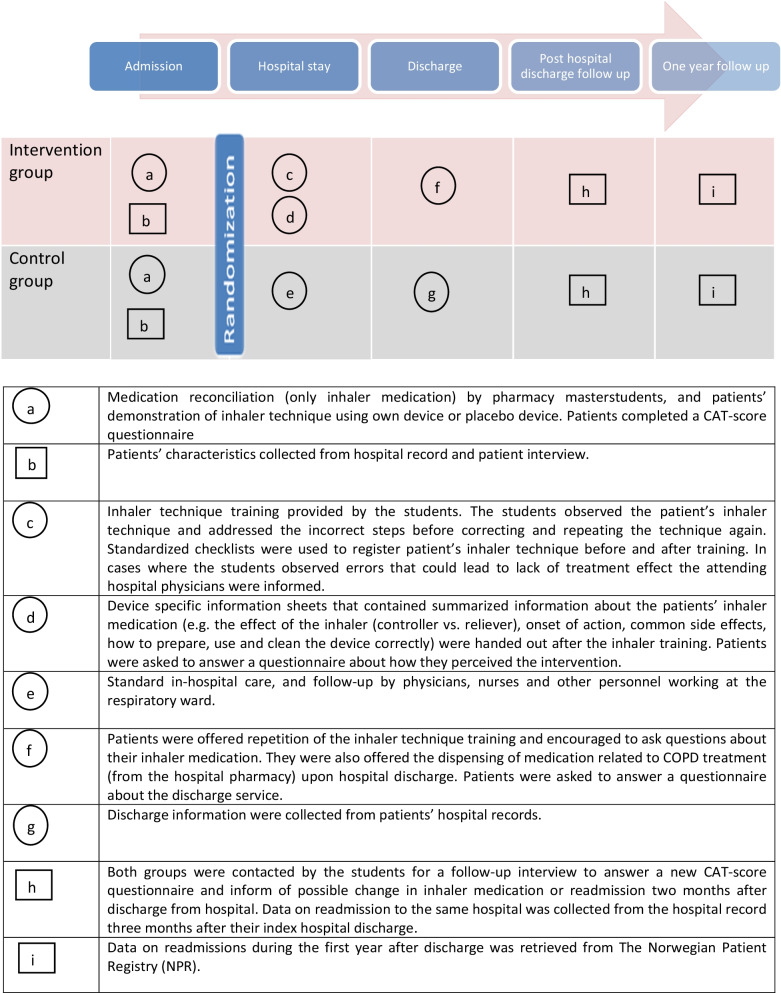
Flow chart showing the different interventions and time course of the pilot study, inspired by Perera et al. [55]. Objects are represented by squares to reflect their fixed nature. Activities are represented by circles to reflect their flexibility. Intervention components delivered consecutively are shown one beneath the other

The master students conducted a medication reconciliation based on the Integrated Medicines Management (IMM) model [46] for the inhaler medications, before assessing the patients’ inhaler technique, Fig. 2. The technique was assessed for all inhalers in use for all patients, by means of a standardized device-specific checklist [33]. Similar checklists were developed for Twisthaler, Easyhaler and Aerochamber, not included in the previous study. For each checklist, some steps were defined as critical [33]. The inhaler technique was categorized as ‘’optimal’’ (all steps performed correctly), ‘’acceptable’’ (all critical steps performed correctly), or ‘’inadequate’’ (some or all critical steps performed incorrectly).

The COPD Assessment Test (CAT) was performed preferably during the first day of hospital admission using the CAT-score questionnaire [9], Fig. 2. The questionnaire comprised eight questions, rated with a number between 0 and 5, with a total score range between 0 and 40. Patients were encouraged to fill in the questionnaire themselves, but the students assisted if needed.

### The intervention group

The intervention group received inhaler technique training during the hospital stay and were offered a second inhaler technique training and dispensing of their COPD medication upon discharge. The discharge service was offered the day before or on the day of hospital discharge.

Prior to study start, the master students received training in performing correct inhalation technique checks from an experienced pharmacist with an inhalation-training licence. The inhaler technique training was based on a standardized procedure developed by The Norwegian Pharmacy Association [47]. Patients demonstrated their inhaler technique to the master students, who addressed the incorrect steps and then demonstrated the correct inhalation technique for the patients. Device-specific information sheets were developed and then approved by the chief physician at the ward, before they were handed out to patients after the training. The information sheets contained summarized information about the inhaler medication, e.g. the effect (controller vs. reliever), onset of action, common side effects, and how to prepare, use and clean the device correctly. If the master students observed critical errors that could lead to a lack of treatment effect, the attending hospital physicians were informed. The intervention included the master students suggesting a change in COPD treatment like adding or discontinuing an inhaler medication or changing the inhaler device when deemed appropriate based on treatment guidelines. The decision to implement the suggested changes was made by a physician.

In addition to the CAT-score questionnaire, patients were asked to answer two other questionnaires; one about their experience with the inhalation training during the hospital stay, based on the study by Ruud et al. [33]. The other questionnaire concerned the medicine dispensing at discharge service, and was developed and adjusted after input from a user representative. The master students asked the questions and noted down patients’ answers. To reduce response bias these interviews were conducted by the student who had not delivered the intervention to the individual patient.

### The control group

The control group received the standard care at the ward, without further contact with the master’s students beyond the baseline assessments. The hospital physicians were informed if critical errors that could lead to a lack of treatment effect were observed at baseline.

### Follow-up

Patients in both groups were contacted by the master`s students for a follow-up phone interview 2 months following hospital discharge. The interview focused on assessing the patients’ current CAT-score. Data on readmissions 3 months after the patient`s index hospital discharge was collected from the hospital record, and data on readmissions during the first year after discharge was retrieved from The Norwegian Patient Registry (NPR).

### Outcomes

Primary outcome measure:

- Difference in time to first readmission after hospital discharge between the treatment groups.
  Secondary outcome measures:
- Proportion of patients readmitted within 90 days and 1 year after hospital discharge in the intervention versus the control group.
- Difference in CAT-score 2 months after hospital discharge between the two groups.
- Patients’ perception of the usefulness of pharmacist-led inhaler technique training and inhaler medication dispensing upon hospital discharge.

### Sample size

The sample size calculation was based on the primary outcome of a study that measured the proportion of patients readmitted after 90 days, assuming that the «care coordination»- group in that study could be considered similar to the intervention group in our study [43]. To detect a difference in readmission rate between the groups, with a significance level of 5% and power of 80%, a sample size of 40 patients was needed. Calculations based on proportions are generally considered reliable for survival analysis, but might overestimate the required sample size [48].

### Randomization and blinding

Patients were randomized by permuted blocks, block sizes varying between 4 and 6. The randomization sequence was generated by R (R Foundation for Statistical Computing). Author LM, who did not recruit and allocate patients, prepared opaque, sealed, and sequentially numbered envelopes containing the randomization codes. The master students assigned the envelope with the lowest number to the individually enrolled patient and signed the allocation after the envelope was opened.

It was not possible to blind the investigators, or the staff at the ward to the patients’ group allocation as the intervention included suggesting a change in COPD treatment. The baseline assessments were performed before the patient was allocated to the treatment group.

### Data analysis

The primary endpoint was analysed by the Kaplan–Meier method and the log-rank test. Cox’s proportional hazards model was applied to estimate hazard ratios (HRs), with corresponding 95% confidence intervals (CIs). The proportionality assumption was checked by visual inspection of log(− log) plots. Data were analyzed according to the intention to treat (ITT) principle. Data were registered using a database in Epidata Entry Client®, version 4.2.0. The data were exported and analysed with the Statistical Package for Social Sciences (SPSS) version 25.

Categorical variables were presented as proportions, and continuous variables as means with standard deviations (SD), or medians with ranges. The difference in proportion of readmitted patients between treatment groups, as well as the difference in inhaler technique before and after inhaler training in the intervention group, were compared using the Fisher’s exact test. The difference in CAT-score 2 months after hospital discharge was compared using Mann–Whitney U test. *p* values < 0.05 were considered statistically significant.

## Results

### Study population

A total of 129 COPD patients were evaluated for eligibility, whereof 72 (56%) were invited to participate, and 40 provided written informed consent and were enrolled into the study. One patient in the intervention group withdrew their consent after randomization due to worsened health conditions, but gave permission to the use of the data collected in the baseline analysis. Thus, the analysis population for the primary endpoint comprised 19 intervention and 20 control patients, Fig. 1.

The mean age of the included patients was 73.8 ± 8.2 years, Table 1. Study subjects were hospitalized on average 2.2 ± 2.7 times within the 12 months prior to the index admission, whereof 1.3 ± 2.0 hospitalizations were caused by COPD exacerbations. Patients in the intervention group seemed to have a higher number of comorbidities and experienced more exacerbations in the previous 12 months than patients in the control group. COPD exacerbation, with or without infection, was the most common cause of hospital admissions, in total, 18 patients.

**Table 1:** Patients’ baseline characteristics

Characteristics	Intervention group (20 patients)	Control group (20 patients)	Total (40 patients)
Age, mean ± SD years			
Female	73.1 ± 9.1	74.4 ± 9.7	73.7 ± 9.2
Male	73.4 ± 7.4	74.5 ± 6.1	74.0 ± 6.5
Sex, *n*			
Female	13	12	25
Male	7	8	15
Number of comorbidities, mean ± SD	4.9 ± 2.7	3.9 ± 2.2	4.4 ± 2.5
Most frequent comorbidities, *n*			
Hypertension	7	7	14
Osteoporosis	6	6	12
Chronic respiratory failure	7	5	12
Coronary heart disease	5	3	8
Congestive heart failure	5	3	8
Atrial fibrillation	6	2	8
Cause of hospitalization at time of recruitment to study: *n*			
COPD exacerbation	13	5	18
COPD exacerbation with infection	4	9	13
COPD exacerbation with lung embolism	0 (0)	1	1
Chronic respiratory failure	0 (0)	1	1
Planned CT-guided biopsy	3	3	6
Sleep apnea test	0 (0)	1	1
Fall	0 (0)	1	1
Number of patients admitted to the hospital** in the last 12 months *n*	17	14	31
Number of hospitalizations in the last 12 months, mean ± SD	3.1 ± 3.4	1.4 ± 1.4	2.2 ± 2.7
Number of hospitalizations caused by COPD exacerbation in the last 12 \months, mean ± SD	1.8 ± 2.5	0.8 ± 1.2	1.3 ± 2.0
Number of hospitalization days at initial admission, mean ± SD	8.2 ± 6,1	6.6 ± 4.5	7.4 ± 5.3
COPD-stage (GOLD-classification)			
Stage 2	2	5	7
Stage 3	8	7	15
Stage 4	6	6	12
ACOS***	1	0	1
Unknown	3	2	5
Number of inhalers, *n*			
One	1	4	5
Two	7	10	17
Three	12	6	18
Number of inhalers device*, *n*			
One	1	4	5
Two	9	11	20
Three	10	5	15
CAT-score median	29	24	24.5

*CAT* COPD Assessment Test

*Most patients used more than one inhaler device each

**The hospital is the patients’ local hospital

***ACOS: Asthma-COPD overlap syndrome

The most commonly used inhalers were aerosols, used by 33 patients (83%). Of these, 17 patients (52%) used the aerosols with an inhalation chamber. Only 24 patients (60%) said that they had previously received inhaler technique training for one or more of their inhalers, and of these 50% said that they received such training during the previous 12 months.

### Readmissions

**Fig. 3:**
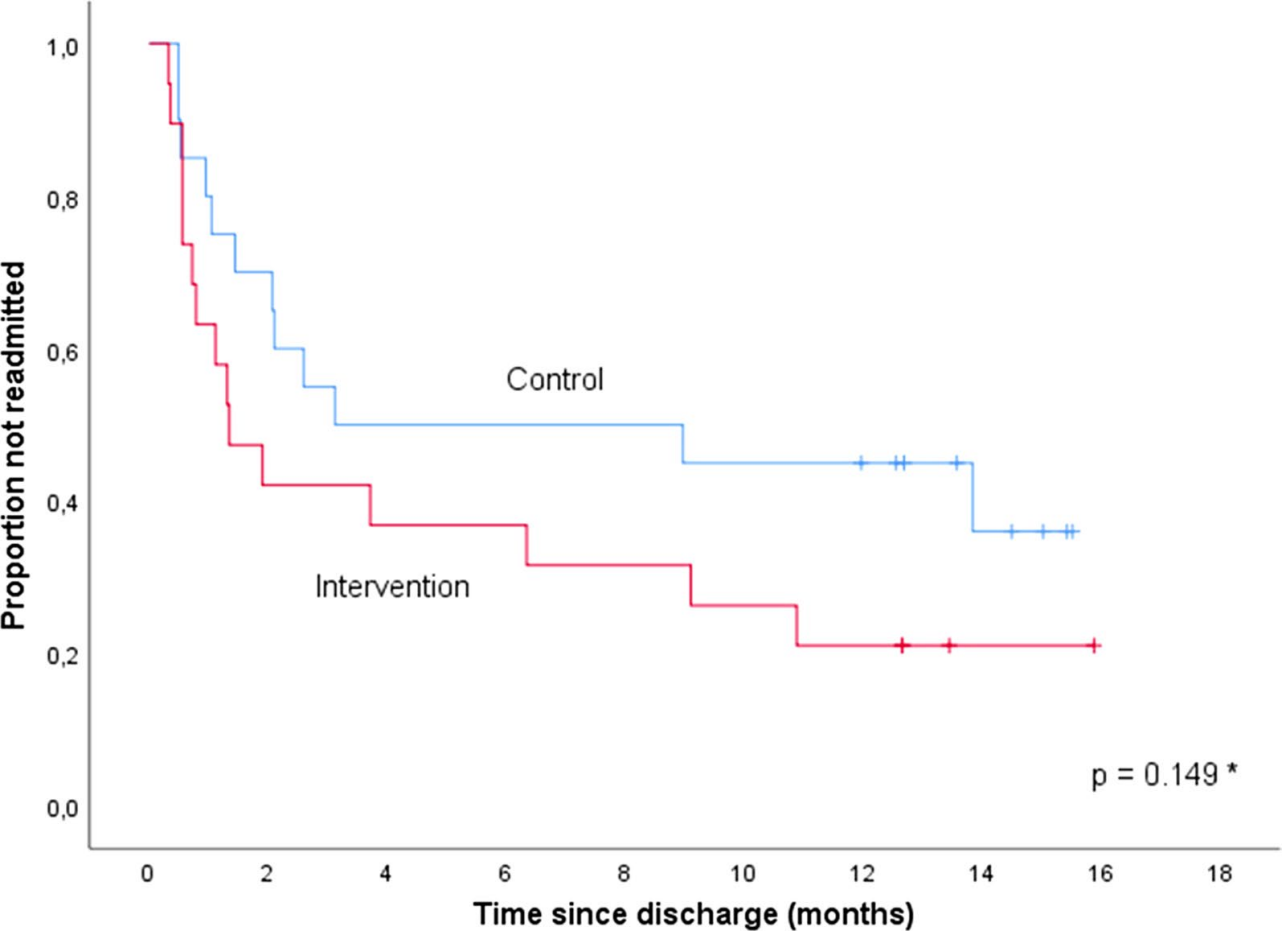
Time to readmission in the intervention versus control group. *Logrank test

The intervention had no significant effect on time to readmission during the 12 months follow-up, Fig. 3. The median time to readmission was 41 days in the intervention group and 95 days in the control group, (HR 1.74, 95% CI 0.81–3.75, *p* = 0.16). A sensitivity analysis adjusting for the duration of index hospital stay and number of hospitalizations 12 months before index admission showed no difference between the two groups (HR 1.15, 95% CI 0.49–2.67, *p* = 0.75).

Eleven patients (58%) in the intervention group and nine (45%) in the control group were readmitted within 90 days, *p* = 0.53. In total 15 patients (79%) in the intervention group and 12 (60%) in the control group were readmitted within 1 year, *p* = 0.30.

COPD exacerbations were the most frequent reason for readmission causing 52% (n = 13) of the readmissions, 8 patients in the intervention group, and 5 in the control group. Twelve patients had the same admission diagnosis (COPD exacerbation) at initial hospitalization and readmission. Other causes of readmission were pneumonia (n = 1), asthma exacerbation (non-infectious) (n = 1), pleural effusion (n = 1), scheduled invasive tests (n = 4), congestive heart failure (n = 1), fall (n = 1), erysipelas (n = 1), chest pain (n = 1), and atherosclerosis (n = 1).

### CAT-score

Forty patients completed the CAT-score at hospital admission, whereof one withdrew the consent (intervention) and three (one intervention, two control) died within 2 months after discharge. Furthermore, seven patients (two intervention, five control) did not complete the CAT questionnaire after discharge. Thus, the analysis population for this endpoint consisted of 29 patients. There was no statistically significant difference between the groups in CAT-score 2 months after hospital discharge, median scores being 25.5 and 24 in the intervention and the control group, respectively (*p* = 0.29). For the 29 patients constituting the analysis population for this endpoint, there was, however, a reduction of 3.5 units in CAT-score from baseline to 2 months after discharge in the intervention group (median 29 at baseline), compared to no change in the control group (median 24 at baseline).

### Inhaler technique

At baseline, 25 patients had inadequate inhaler technique for all inhalers, while 10 had optimal or acceptable inhaler technique.

In total 97 inhalers were registered at baseline, whereof only two (2%) were used with optimal inhaler technique. In comparison, 25 (26%) were used with acceptable technique, and 61 (63%) were used with inadequate technique. Some patients refused to demonstrate their inhaler technique, thus there were nine inhalers (9%) with no demonstrated user technique.

The most frequent error identified during the baseline check was errors during the inhalation, including not using the appropriate inhalation force while inhaling, and inadequate inhalation time. Other common errors were patients not breathing out before using the inhaler, not having the proper body posture, and not holding their breath after inhaling.

The number of inhalers included in the analysis in the intervention group was 48, of which 31 had the technique checked at baseline and directly after the inhaler training. After the training sixteen inhalers (33%) were demonstrated with optimal inhaler technique, nine inhalers (19%) with acceptable, and six inhalers (13%) with inadequate technique. Seventeen inhalers (35%) had missing data about technique after the intervention. The reason being that some patients did not thoroughly demonstrate the inhaler technique after intervention (n = 13), before and after intervention (n = 2), or did not demonstrate their technique at baseline (n = 2). The results showed no significant improvement in inhaler technique (*p* > 0.05) directly after inhaler training compared to the technique before the intervention.

### Discharge service

Nineteen patients were offered dispensing of their prescribed inhalers before discharge, whereof 14 (74%) responded they had no need for this service because they had brought their own inhalers. Only five patients (26%) received their prescribed inhalers at the ward before hospital discharge. The majority of patients (n = 17, 90%) did not want a repetition of the inhaler technique training before discharge. Only two patients (11%) accepted that service.

### Usefulness of study intervention

All 19 patients in the intervention group were invited to evaluate the intervention, and of these 18 completed the questionnaire on inhaler technique training. The majority of the patients (n = 16, 89%) stated that they were satisfied with the inhaler technique training, and 14 patients (78%) perceived the intervention as useful.

Fourteen patients completed the questionnaire on the medication dispensing service, and one patient answered parts of the questionnaire. Eleven patients stated that it is important to be offered medication dispensing upon hospital discharge, and 10 stated that they would make use of the service if offered in the future.

## Discussion and conclusion

### Discussion

The main objective of the pilot study was to provide an effect estimate of an educational inhaler technique intervention on time to first readmission in a population of hospitalized patients with COPD.

However, the intervention had no effect on readmissions, neither on time to first readmission nor proportion of readmitted patients within 3 months and 1 year.

There were some imbalances between the treatment groups at baseline. The intervention group had a higher frequency of hospitalizations during the previous year, a higher number of inhalers, longer hospital stays, a higher number of comorbidities and higher CAT-scores compared to the control group. However, the sensitivity analyses adjusting for the duration of index hospital stay and number of hospitalizations 12 months before index admission did not change the results to any considerable degree.

There was no statistically significant difference in CAT-score between the two groups 2 months after discharge. The assumptions for ANCOVA were not met, preventing an adjustment for baseline values, which is a limitation. However, the reduction in CAT-score between baseline and 2 months after discharge was 3.5 units in the intervention group, compared to no change in the control group According to the CAT Development Steering Group, a change of two or more units over 2–3 months suggests a clinically significant difference or change in health status [49]. Thus, the results may indicate that the CAT score could be explored as a possible clinical endpoint for inhaler technique interventions.

The results differ from previous trials on similar interventions showing decreased hospital readmissions, and improvement in inhaler technique and CAT-score [32–38, 41–43]. The patient population may explain these differences. Our study included elderly (median age 73 years), multimorbid COPD patients, with more severe diagnosis. A predominance of patients were in GOLD group D (68%), and 98% had a CAT-score above 10. Furthermore, the majority of the patients (87%) used several inhalers and approximately half of the patients had been hospitalized due to COPD exacerbations during the last year before index hospitalisation. In this population, frequent hospital readmissions might be difficult to avoid, and it has been shown that the course of COPD involves a rapid decline in health status and a sharp increase in the risk of new exacerbations after the second severe exacerbation [50]. This would suggest that the target population for inhaler technique interventions at hospitals should be patients admitted for their first exacerbations. Further support to this theory is lent by a subgroup analysis of study on the effect of a pharmacist intervention to improve the medical treatment of patients 80 years and older, which found a higher efficacy in preventing emergency department visits in patients using less than five drugs compared to patients using five drugs or more [51]. That is, the intervention was most effective in the less severely ill patients.

There was no statistically significant improvement in inhaler technique directly after inhaler training compared to before the intervention. However, the inhaler technique for the intervention group seemed to improve from 19% with optimal or acceptable technique before training, to 52% after training for the inhalers that were demonstrated both before and after training. Information was missing for 35% of the inhalers after inhaler technique training mainly because the participants refused to demonstrate their technique again after having been shown the correct technique, or they perceived the corrective advice as unproblematic to implement later on their own. Thus, the acceptability of the intervention in this patient group might be questioned. The lack of improvement in inhalation technique after inhaler training in the intervention group could also contribute to explain the apparent lack of effect on the clinical endpoints, compared to the results seen in some previous studies that used teach-to-goal instruction in inhaler technique training [52]. If improvement in inhaler technique after training is chosen as an indicator to reduce exacerbations and hospital admissions in future studies, one should consider screening at baseline and only include participants with inadequate inhaler technique.

In addition, some participants received new inhalers during the hospital stay, thereby lacking inhalation technique at baseline. This is a limitation to the study since the technique for only 65% of inhalers was evaluated before and after training. The missing information hampers the possibility to conclude on whether the intervention was adequately delivered and whether patients’ inhaler technique was improved. This challenge was not described in a study at community pharmacies, reporting an increased proportion of participants with correct inhalation technique from 31% before training to 86% 3 months after training [33].

Almost half of the patients who were approached declined to participate in the study. Previous studies have shown that COPD patients’ cognition might be reduced at the time of admission with an exacerbation, but improve to time of discharge [53, 54]. Thus, it is possible that the recruitment rate could be increased if the time window for inclusion was prolonged, allowing patients to recover somewhat from their acute status at admission.

Stratifying for differences that might cause uneven distribution of baseline variables, for example frailty, CAT-score, or previous hospital admissions, could have been useful when studying the effect of pharmacist-led inhalation training on COPD patients at a high risk of readmission. Nonetheless, the inclusion criteria resulted in the inclusion of a multimorbid COPD patient population, for whom frequent hospital readmissions due to exacerbations and other health issues might be difficult to avoid. We did not have permission to gather detailed data about the patients who died. Therefore, we were not able to censor for death in the survival analysis, which is a limitation. Another limitation is the fact that the hospital physicians, for ethical reasons, were informed of critical errors that could potentially cause lack of effect in the control group. This could have reduced the chance of observing true treatment effects.

Studying CAT-score as a potentially sensitive measurement for clinical effect of inhalation training among hospitalized patients with severe COPD might be a better strategy. However, measuring CAT-score after discharge was challenging and resulted in a high number of drop-outs. If CAT-score after discharge is chosen as an endpoint in future studies, it is crucial to account for a high number of drop-outs in the sample size calculation.

In general, the patients responded positively to receiving inhalation technique training. Despite not using the service when offered, the patients were also positive to the inhaler technique training repetition and medication dispensing service upon discharge. Participants' reasoning for declining the discharge services were not systematically collected and registered, but the most common reason given by participants for not repeating the inhalation technique training upon discharge was that they felt confident about the technique after the first round with inhaler training and were in no need for repetition. While other participants said that they were more focused on getting ready to be discharged and wanted to rest. Therefore, we were not able to observe whether the inhalation technique was improved upon hospital discharge, which is a limitation. Future studies should take this into consideration and coordinate the discharge services with the physicians involved in discharging the patients.

### Conclusion

The pharmacist-led inhalation technique training had no statistically significant effect on time to readmission and CAT-score for hospitalized, multimorbid COPD patients, thus the study did not contribute data for sample size calculations for future studies. Future studies in larger populations should consider to focus on patients with less severe COPD, exploring the CAT-score as a possible primary endpoint, consider stratifying for important baseline variables and evaluate the acceptability of the intervention in the study population. If CAT-score after discharge is chosen as an endpoint in future studies, the sample size calculation should account for a high number of drop-outs.

## Data Availability

The dataset supporting the conclusions of this article is included within the article and its additional files.
